# Emergence delirium is associated with increased postoperative delirium in elderly: a prospective observational study

**DOI:** 10.1007/s00540-020-02805-8

**Published:** 2020-06-07

**Authors:** Yan Zhang, Shu-Ting He, Bin Nie, Xue-Ying Li, Dong-Xin Wang

**Affiliations:** 1 Departments of Anesthesiology and Critical Care Medicine, Peking University First Hospital, No.8 Xishiku Street, Beijing, 100034 China; 2 Departments of Anesthesiology, Fujian Provincial Cancer Hospital, Fuzhou, Fujian China; 3 Department of Biostatistics, Peking University First Hospital, Beijing, China; 4 Department of Outcomes Research Consortium, Cleveland Clinic, Cleveland, OH USA

**Keywords:** Elderly, General anesthesia, Major surgery, Emergence delirium, Postoperative delirium

## Abstract

**Background:**

The clinical significance of emergence delirium remains unclear. The purpose of this study was to investigate the association between emergence delirium and postoperative delirium in elderly after general anesthesia and surgery.

**Methods:**

This prospective observational study was done in a tertiary hospital in Beijing, China. Elderly patients (65–90 years) who underwent major noncardiac surgery under general anesthesia and admitted to the postanesthesia care unit (PACU) after surgery were enrolled. Emergence delirium was assessed with the Confusion Assessment Method for the Intensive Care Unit during PACU stay. Postoperative delirium was assessed with the Confusion Assessment Method during the first 5 postoperative days. The association between emergence delirium and postoperative delirium was analyzed with a multivariable logistic regression model.

**Results:**

A total of 942 patients were enrolled and 915 completed the study. Emergence delirium developed in 37.0% (339/915) of patients during PACU stay; and postoperative delirium developed in 11.4% (104/915) of patients within the first 5 postoperative days. After adjusted confounding factors, the occurrence of emergence delirium is independently associated with an increased risk of postoperative delirium (OR 1.717, 95% CI 1.078–2.735, *P *= 0.023). Patients with emergence delirium stayed longer in PACU and hospital after surgery, and developed more non-delirium complications within 30 days.

**Conclusions:**

Emergence delirium in elderly admitted to the PACU after general anesthesia and major surgery is independently associated with an increased risk of postoperative delirium. Patients with emergence delirium had worse perioperative outcomes.

*Chinese Clinical Trial Registry (chictr.org.cn)* ChiCTR-OOC-17012734

**Electronic supplementary material:**

The online version of this article (10.1007/s00540-020-02805-8) contains supplementary material, which is available to authorized users.

## Introduction

Delirium is a common complication in elderly patients after surgery [1]. According to the onset time, delirium in the postoperative period can be classified as emergence delirium and postoperative delirium (POD). Emergence delirium occurs during or immediately after emergence from general anesthesia; whereas POD is usually notable from postoperative day 1 and up to 1 week after surgery [2, 3]. The reported incidence of the two types of delirium varies depending on patient population, type of anesthesia/surgery, and assessment methods. Indeed, emergence delirium occurs in 3.7–45% of patients [4–15] and the reported rate of POD is 5–54% after noncardiac surgeries [16–18].

The underlying mechanisms of POD remain unclear but multi-risk factors include predisposing and precipitating factors are associated with its development [1]. The development of POD is associated with worse outcomes such as more complications, longer hospital stays, higher medical expense, as well as shortened overall survival and lowered quality of life [19–21]. In contrast, it was considered that emergence delirium is likely related to the effects of residual general anesthetics and is “self-limited” without sequelae [2, 4]. However, recent studies suggested that emergence delirium shares some common risk factors with POD, and that its occurrence is also associated with worse perioperative outcomes including more pulmonary complications, longer length of hospital stay and high readmission rate [5, 8–10, 14, 15, 22].

Studies of emergence delirium in the elderly are inadequate and the clinical significance of emergence delirium remains poorly understood. We hypothesize that elderly with emergence delirium would have a higher risk of postoperative delirium and worse outcomes. The primary purpose of this study was to investigate the association between emergence delirium and POD in elderly after general anesthesia and major surgery.

## Methods

### Study design

This prospective observational study was carried out in a tertiary hospital in Beijing, China. The study protocol was approved by the Clinical Research Ethics Committee of Peking University First Hospital on August 4, 2017 (2017[1419], Beijing, China) and was registered with Chinese Clinical Trial Registry on September 19, 2017 (chictr.org.cn, ChiCTR-OOC-17012734). Written informed consent was obtained from all participants or their legal representatives.

### Participants

The inclusion criteria were patients aged 65–90 years who were scheduled to undergo major noncardiac surgery with an expected duration ≥ 2 h under general anesthesia and admitted to the post-anesthesia care unit (PACU) after surgery. Patients who met any of the following criteria were excluded: (1) refused to participate in the study; (2) previous history of schizophrenia, epilepsy, Parkinson’s Disease, or myasthenia gravis; (3) unable to communicate due to severe dementia, comatose or language barrier; (4) traumatic brain injury or neurosurgery; or (5) an American Society of Anesthesiologists (ASA) classification of IV or above.

### Anesthesia and perioperative care

Monitoring in the operating room includes electrocardiogram, non-invasive blood pressure, pulse oxygen saturation, end-tidal carbon dioxide, expired concentration of inhalational anesthetics, bispectral index, and urine output. Invasive arterial pressure and central venous pressure were monitored when considered necessary. General anesthesia was induced with propofol and/or etomidate, opioids (sufentanil and/or remifentanil) and muscle relaxants (rocuronium or cisatracurium). Airway was secured with a laryngeal mask or an endotracheal tube, depending on the type and estimated length of surgery as well as patients’ position during surgery. Anesthesia was maintained with propofol infusion and/or sevoflurane inhalation, with or without nitrous oxide. Opioids and muscle relaxants were administered when considered necessary. Nonsteroidal anti-inflammatory drugs were administered for those without contraindications at the discretion of anesthesiologists. The target was to maintain bispectral index between 40 and 60.

Before the end of surgery, muscle relaxants were stopped for at least 30 min; propofol infusion and/or sevoflurane inhalation were decreased or stopped according to BIS monitoring; sufentanil was administered when considered necessary. At the end of surgery, residual neuromuscular blockade was reversed with 0.05 mg/kg neostigmine and 0.02 mg/kg atropine. Patients were extubated when they met the following criteria: (1) easy to wake up; (2) sufficient reflexes that protect the airway; (3) adequate gas exchange (respiration rate 10–30 breaths per minute and tidal volume > 6 ml/kg); and (4) acceptable hemodynamic status (systolic blood pressure ≥ 90 mmHg and heart rate ≤ 100 beats per minute). As a routine practice, patients were extubated in the operating room and were transferred to the PACU before being sent back to the wards.

Patients were monitored in PACU for at least 30 min. Routine monitoring included electrocardiogram, non-invasive blood pressure, pulse oxygen saturation. Pain severity was assessed with the numeric rating scale (NRS, an 11-point scale where 0 = no pain and 10 = the worst pain). Moderate to severe pain (NRS pain score > 3) was managed with intravenous opioids and/or non-steroid anti-inflammatory drugs (NSAIDs). Tympanic temperature was measured with an infrared ear thermometer. Patients with hypothermia (< 36 °C) were managed with warm air blanket. Supplemental oxygen was provided. Patients were transferred to the general ward when they met all of the following criteria: (1) full consciousness; (2) able to lift head for more than 10 s; (3) able to keep airway clear and normal ventilation, pulse oxygen saturation > 95% in room air for more than 5 min; (4) stable circulation, with heart rate and blood pressure within 20% from baseline without vasoactive drugs; and (5) no anesthesia- or surgery-related complications. In the general wards, patients were monitored intermittently for pulse oxygen saturation and non-invasive blood pressure until next morning.

### Data collection and postoperative follow-ups

Data collection was performed after obtaining written informed consents. Baseline data included demographics, education background, surgical diagnosis, comorbidities, preoperative medication, smoking and alcoholism, Charlson Comorbidity Index [23], laboratory test results, and ASA classification. Baseline cognitive function was evaluated with the Mini-Mental State Examination (MMSE, scores range from 0 to 30, with higher scores indicating better function) before surgery. Intraoperative data included type of anesthesia, types and doses of anesthetic drugs, intraoperative liquid balance (including estimated blood loss and blood transfusion), type and location of surgery, as well as duration of anesthesia and surgery. Emergence agitation in the operating room was assessed by the attending anesthesiologists using the Richmond Agitation Sedation Scale (RASS; scores range from –5 [unarousable] to +4 [combative] and 0 indicates alert and calm) [24]. A RASS score of ≥+1 was defined as having emergence agitation.

Postoperative data during PACU stay included NRS pain score, tympanic temperature, usage of analgesics, occurrence of delirium, occurrence of other adverse events/complications and management, and length of PACU stay. In the general wards, patients were followed up twice daily until the 5th day after surgery for the occurrence of delirium, the severity of pain, usage of analgesics, and the occurrence of other complications.

Prior to the study period, investigators who performed delirium assessment (YZ, STH and BN) were trained to use the Confusion Assessment Method (CAM) [25] and the CAM for the Intensive Care Unit (CAM-ICU) [26] by a psychiatrist (XYS). Both CAM and CAM-ICU detect four features of delirium, i.e., (1) acute onset of mental status changes or a fluctuating course, (2) inattention, (3) disorganized thinking, and (4) altered level of consciousness. To have delirium diagnosed, a patient must display features 1 and 2, with either 3 or 4 [25, 26]. The training program included lectures introducing delirium and the CAM/CAM-ICU, as well as simulation courses with patient-actors. The initial training continued until the diagnosis of delirium reached 100% agreement with the psychiatrist. The training process was repeated at least two times a year. During the study period, investigators who performed delirium assessment did not participate in perioperative care of the enrolled patients. We have used these instruments in our previous studies and have considerable experience with the techniques [27, 28].

In the present study, emergence delirium was defined as delirium that occurred during PACU stay and was assessed with the CAM-ICU at 10 and 30 min after PACU admission, and before PACU discharge. Immediately before assessing delirium assessment, the level of sedation/agitation was evaluated with the RASS. If the patient was deeply sedated or unarousable (−4 or -5 on the RASS), delirium assessment was stopped and the patient was noted as comatose. If the RASS was above −4 (−3 through +5), delirium assessment was performed. Emergence delirium was classified into 3 subtypes, i.e., hyperactive (with a consistently positive RASS, from +1 to +4), hypoactive (with a consistently neutral or negative RASS, from 0 to −3) and mixed [29]. Postoperative delirium was defined as delirium that occurred in the general wards during postoperative days 1 to 5 and was assessed with the CAM twice daily (8:00–10:00 am, 18:00–20:00 pm). Pain severity was assessed with the NRS at the same time interval as that of delirium.

From the 6th day after surgery, patients were followed up weekly until postoperative day 30 for the occurrence of postoperative complications. For those who were discharged from the hospital, follow-ups were performed by telephone interview. Non-delirium complications were generally defined as any new onset medical conditions that adversely affect patients’ recovery and require medical intervention, i.e., grade II or higher on the Clavien-Dindo classification [30, 31]. On postoperative day 30, quality of life was evaluated with the World Health Organization Quality of Life-brief version (WHOQOL-BREF, scores range from 0 to 100, with higher score indicating better quality of life) [32]; cognitive function was evaluated with the Chinese version of Telephone Interview for Cognitive Status-modified (m-TICS, scores range from 0 to 50, with higher score indicating better cognitive function) [33].

The primary outcome was the occurrence of emergence delirium, i.e., delirium that developed during PACU stay. Secondary outcomes included the incidence of postoperative delirium, the length of PACU and hospital stay after surgery, the occurrence of non-delirium complications within 30 days, and the cognitive function and quality of life in 30-day survivors.

## Statistical analysis

### Estimation of sample size

According to published data and our own results, we assumed that the incidence of emergence delirium and POD was 23.5% and 8%, respectively. Sample size was estimated with the following formula:

$$n\, = \,\frac{{Z_{1 - \alpha /2\,}^{2} \,V\left( {\mathop \vartheta \limits^{{ \wedge }} } \right)}}{{L^{2} }}$$

$V\left( {\mathop \vartheta \limits^{{ \wedge }} } \right)$ (variance function) is estimated with the sensitivity (Se):$V\left( {\mathop \vartheta \limits^{{ \wedge }} } \right)\, = \,Se\, \times \,\left( {1 - Se} \right)$. L is half-width of confidence interval for clinical acceptance.

With the significance level (α) set at 0.05, sensitivity (Se) at 80%, and half-width of confidence interval at 0.1, respectively, a total of 62 cases with positive events was needed. Considering a POD incidence of 8% and possible drop-out, 942 patients were enrolled in the present study.

### Data analysis

Patients were divided into two groups, i.e., those with emergence delirium and those without. Continuous variables with normal distribution were analyzed with unpaired *t* test. Continuous variables with non-normal distribution or ordinal data were analyzed with Mann–Whitney U test. Categorical variables were analyzed with Chi squared test, continuity correction Chi squared test or Fisher exact test. Time-to-event results were analyzed using the Kaplan–Meier survival analysis, with the difference between groups tested by the log-rank test. Univariable associations between baseline and perioperative variables and POD were performed with logistic regression analyses. To assess the association between emergence delirium and POD, factors with *P* < 0.20 in univariable analyses and those that were considered clinically important were included in a multivariable logistic regression model to adjust the effects of confounding factors. Data analyses were performed with the SPSS 25.0 software (IBM SPSS Inc, Chicago, IL). A two-sided *P* < 0.05 was considered statistically significant.

## Results

### Patient population

**Fig. 1:**
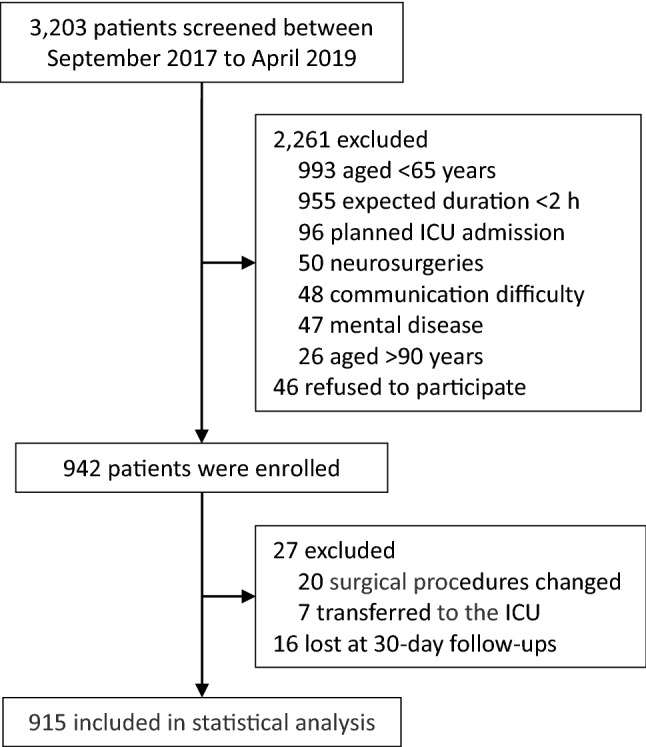
Flowchart of the study

From September 21, 2017 to April 10, 2019, 942 patients who met the inclusion/exclusion criteria and gave consents and were enrolled into the study. During the study period, 27 patients were excluded due to changed surgical procedures or ICU admission after surgery. At last, 915 patients were included in the final analysis (Fig. 1).

### Occurrence of emergence delirium

**Fig. 2:**
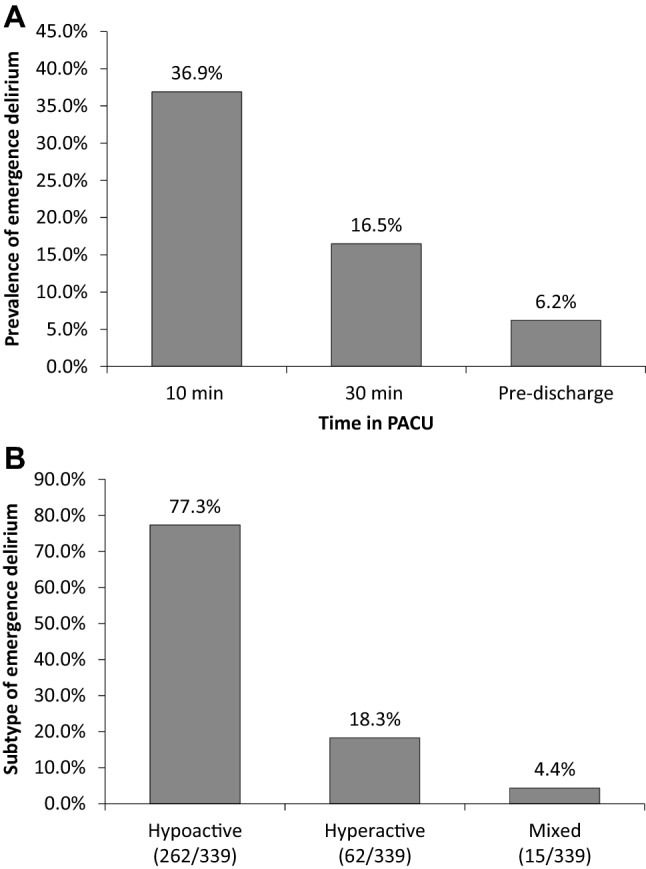
Prevalence (A) and motoric subtype (B) of emergence delirium after surgery

Of all included patients, 37.0% (339/915) developed emergence delirium during PACU stay. The prevalence of emergence delirium was the highest at 10 min after PACU admission and gradually decreased along with time; 77.3% (262/339) of emergence delirium was hypoactive subtype (Fig. 2). Before surgery, patients who developed emergence delirium were older, received shorter education, and had more coronary heart disease, previous surgery and higher ASA grade; but they had lower hematocrit and albumin levels as well as lower MMSE score. During the intra- and postoperative period, patients who developed emergence delirium were given more etomidate and propofol, underwent more non thoraco-/laparoscopic but less intra-thoracic surgeries, lost more blood, received more fluid infusion and blood transfusion, developed more emergence agitation, and had more hypothermia and more severe pain during PACU stay (Table 1).

**Table 1:** Baseline and perioperative data

	Total (*n* = 915)	Emergence delirium (*n* = 339)	No emergence delirium (*n* = 576)	*P* value
Age (year)	71.6 ± 5.2	72.9 ± 5.7	70.9 ± 4.8	<0.001
Male sex	548 (59.9%)	192 (56.6%)	356 (61.8%)	0.123
Body mass index (kg/m^2^)	24.2 ± 3.5	24.0 ± 3.7	24.2 ± 3.3	0.473
Education (year)	10.2 ± 4.6	9.4 ± 4.4	10.6 ± 4.7	<0.001
Preoperative comorbidity				
Stroke^a^	52 (5.7%)	16 (4.7%)	36 (6.3%)	0.336
Hypertension	475 (51.9%)	173 (51%)	302 (52.4%)	0.683
Coronary heart disease	129 (14.1%)	58 (17.1%)	71 (12.3%)	0.046
Arrhythmia	57 (6.2%)	24 (7.1%)	33 (5.7%)	0.415
Pulmonary disease^b^	66 (7.2%)	30 (8.8%)	36 (6.3%)	0.144
Diabetes	219 (23.9%)	84 (24.8%)	135 (23.4%)	0.646
Abnormal liver function^c^	45 (4.9%)	19 (5.6%)	26 (4.5%)	0.462
Malignant tumor	105 (11.5%)	39 (11.5%)	66 (11.5%)	0.983
Chronic smoking^d^	223 (24.4%)	71 (20.9%)	152 (26.4%)	0.064
Alcoholism^e^	144 (15.7%)	45 (13.3%)	99 (17.2%)	0.116
Charlson Comorbidity Index^f^	2 (2, 3)	2 (2, 3)	2 (2, 3)	0.580
History of surgery	491 (53.7%)	199 (58.7%)	292 (50.7%)	0.019
ASA classification				0.018
Class II	678 (74.0%)	236 (69.6%)	442 (76.7%)	
Class III	237 (26.0%)	103 (31.4%)	134 (23.3%)	
Preoperative lab tests				
Hematocrit (%)	39.4 ± 5.3	38.5 ± 5.8	39.9 ± 4.9	<0.001
Albumin (g/L)	40.6 ± 4.7	39.9 ± 4.7	40.9 ± 4.7	0.002
Creatinine (μmol/L)	80.2 ± 20.9	81.0 ± 21.9	79.7 ± 20.2	0.354
Glucose < 4.0 or > 10.0 mmol/L	47 (5.1%)	20 (5.9%)	27 (4.7%)	0.423
Na^+^<135.0 or > 145.0 mmol/L	37 (4.0%)	13 (3.8%)	24 (4.2%)	0.802
K^+^ < 3.5 or > 5.5 mmol/L	117 (12.8%)	48 (14.2%)	69 (12%)	0.346
Preoperative MMSE (score)	26.3 ± 2.3	25.5 ± 2.5	26.7 ± 2.1	<0.001
Duration of anesthesia (min)	279 ± 77	285 ± 74	276 ± 79	0.075
Type of anesthesia				0.616
General	420 (45.9%)	161 (47.5%)	259 (45.0%)	
Peripheral-general	469 (51.3%)	167 (49.3%)	302 (52.4%)	
Epidural-general	26 (2.8%)	11 (3.2%)	15 (2.6%)	
Intraoperative medication				
Use of nitrous oxide	553 (60.4%)	212 (62.5%)	341 (59.2%)	0.319
Use of sevoflurane	287 (31.4%)	115 (33.9%)	172 (29.9%)	0.201
Use of dexmedetomidine	430 (47.0%)	164 (48.4%)	266 (46.2%)	0.520
Use of midazolam	189 (20.7%)	65 (19.2%)	124 (21.5%)	0.396
Use of etomidate	699 (76.4%)	273 (80.5%)	426 (74.0%)	0.024
Propofol (mg)	840 (642, 1075)	866 (678, 1132)	824 (622, 1050)	0.048
Remifentanil (μg)	726 (0, 1260)	700 (0, 1230)	760 (0, 1299)	0.819
Sufentanil (μg)	40 (25, 69)	40 (25, 70)	40 (25, 68)	0.391
Sufentanil equivalent (μg)	110 (77, 160)	110 (79, 156)	110 (75, 165)	0.853
Rocuronium (mg)	42.1 ± 19.0	42.2 ± 17.8	42.0 ± 19.7	0.914
NSAIDs^g^	726 (79.3%)	272 (80.2%)	454 (78.8%)	0.609
Duration of surgery (min)	203 ± 71	208 ± 67	199 ± 72	0.078
Type of surgery				0.001
Thoraco-/laparoscopic	549 (59.9%)	179 (52.8%)	369 (64.1%)	
Non thoraco-/laparoscopic	367 (40.1%)	160 (47.2%)	207 (35.9%)	
Location of surgery				0.063
Intra-thoracic	198 (21.6%)	61 (18.0%)	137 (23.8%)	
Intra-abdominal	530 (57.9%)	199 (58.7%)	331 (57.5%)	
Spinal/extremities/others	187 (20.4%)	79 (23.3%)	108 (18.7%)	
Estimated blood loss (ml)	100 (10, 250)	100 (10, 300)	60 (10, 200)	0.002
Lowest hemoglobin < 10 (g/dL)	77 (8.4%)	44 (13.0%)	33 (5.7%)	<0.001
Total fluid infusion (ml)	2200 (1600, 2850)	2350 (1800, 3100)	2100 (1600, 2600)	<0.001
Crystalloid (ml)	1800 (1500, 2300)	2000 (1600, 2500)	1700 (1300, 2300)	<0.001
Artificial colloid	530 (57.9%)	217 (64.0%)	313 (54.3%)	0.004
Artificial colloid (ml)	500 (0, 500)	500 (0, 500)	500 (0, 500)	<0.001
Autologous blood transfusion	104 (11.4%)	48 (13.2%)	56 (9.7%)	0.042
Allogeneic blood transfusion	79 (8.6%)	43 (12.7%)	36 (6.3%)	0.001
Urine output (ml)	400 (250, 600)	400 (250, 700)	400 (250, 600)	0.072
Emergence agitation	99 (10.8%)	73 (21.5%)	26 (4.5%)	<0.001
Temperature in PACU (°C)				
At PACU admission	36.1 ± 0.4	36.0 ± 0.4	36.1 ± 0.4	<0.001
Temperature < 36 °C	301 (32.9%)	154 (45.4%)	147 (25.5%)	<0.001
NRS pain score in PACU				
First pain score	2.0 (2.0, 3.0)	2.0 (2.0, 4.0)	2.0 (1.0, 3.0)	0.003
Average pain score	2.0 (1.3, 3.0)	2.3 (1.7, 3.7)	2.0 (1.0, 3.0)	<0.001
Patient-controlled analgesia				0.611
None	42 (4.6%)	13 (3.8%)	29 (5.0%)	
Intravenous	847 (92.6%)	315 (92.9%)	532 (92.4%)	
Epidural	26 (2.8%)	11 (3.2%)	15 (2.6%)	
Supplemental analgesia in 5 days				
Opioids^h^	133 (14.5%)	55 (16.2%)	78 (13.5%)	0.267
NSAIDs^i^	668 (73.0%)	245 (72.3%)	423 (73.4%)	0.701
NRS pain score in wards				
First pain score	2.0 (1.0, 3.0)	2.0 (1.0, 3.0)	2.0 (1.0, 3.0)	0.785
Average pain score	1.3 (0.3, 2.5)	1.2 (0.2, 2.5)	1.5 (0.3, 2.5)	0.562

Data are mean ± SD, number (%), or median (interquartile range)

*ASA* American Society of Anesthesiologists, *MMSE* mini-mental state examination, *PACU* post-anesthesia care unit, *NRS* numeric rating scales, *NSAIDs* non-steroidal anti-inflammatory drugs

^a^Includes hemorrhagic and ischemic stroke

^b^Includes chronic obstructive pulmonary disease and asthma

^c^Alanine transaminase and/or aspartate transaminase higher than 5 times the upper normal limit

^d^Smoking half a pack of cigarettes per day for at least 2 years

^e^Two drinks or more daily, or weekly consumption of the equivalent of 150 mL of alcohol

^f^According to the Charlson Comorbidity Index without age [23]

^g^Includes parecoxib and flurbiprofen axetil

^h^Includes morphine, oxycodone, and tramadol

^i^Includes parecoxib, flurbiprofen axetil, loxoprofen, and celecoxib

### Effects of emergence delirium on postoperative outcomes

**Fig. 3:**
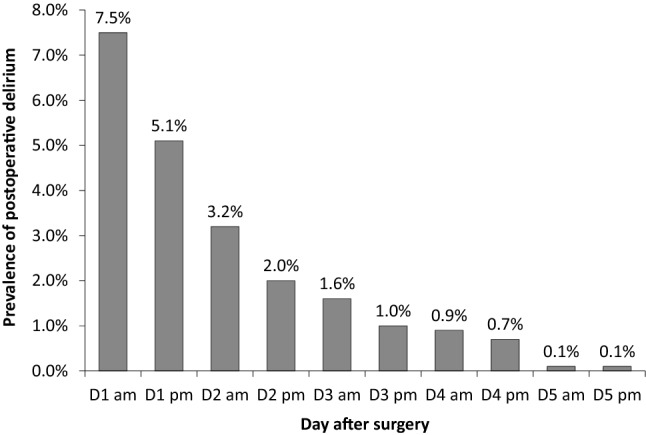
Prevalence of postoperative delirium

Of all included patients, 11.4% (104/915) developed POD within 5 days. The prevalence of POD was the highest in the morning of the first postoperative day, and then gradually decreased along with time (Fig. 3). The incidence of POD was higher in patients with emergence delirium than in those without (16.8% [57/339] vs. 8.2% [47/576]; odds ratio [OR] 2.275, 95% CI 1.506–3.436, *P* < 0.001). Regarding other outcomes, patients with emergence delirium stayed longer in PACU, developed more non-delirium complications within 30 days, and stayed longer in hospital after surgery (Table 2; Supplemental Tables 1 and 2).

**Table 2:** Postoperative outcomes

	Total (*n* = 915)	Emergence delirium (*n* = 339)	No emergence delirium (*n = *576)	OR, mean difference or HR (95% CI)^a^	*P* value
Delirium within 5 days	104 (11.4%)	57 (16.8%)	47 (8.2%)	OR = 2.275 (1.506, 3.436)	<0.001
Length of stay in PACU (min)	43 ± 15	45 ± 16	41 ± 14	Mean D = 4.1 (2.0, 6.1)	<0.001
Length of stay in PACU ≥ 60 min	105 (11.5%)	51 (15.0%)	54 (9.4%)	OR = 1.712 (1.137, 2.576)	0.010
Non-delirium complications within 5 days	75 (8.2%)	35 (10.3%)	40 (6.9%)	OR = 1.543 (0.959, 2.481)	0.074
Non-delirium complications within 30 days	89 (9.7%)	43 (12.7%)	46 (8.0%)	OR = 3.486 (1.296, 9.376)	0.013
Length of stay in hospital (day)	8.0 (7.7, 8.3)	8.0 (7.5, 8.5)	7.0 (6.6, 7.3)	HR = 1.022 (1.003, 1.041)	0.008
Length of stay in hospital ≤ 5 (day)	209 (22.8%)	63 −(18.6%)	146 (25.3%)	OR = 0.672 (0.482, 0.937)	0.019
Length of stay in hospital ≥ 11 (day)	248 (27.1%)	105 (31.0%)	143 (24.8%)	OR = 1.359 (1.009, 1.830)	0.044
Cognitive function at 30 days (score)^b^	34.4 ± 2.4 [16]	34.3 ± 2.4 [7]	34.4 ± 2.4 [9]	Mean D = 0.06 (−0.27, 0.39)	0.736
Quality of life at 30 days (score)^c^					
Social relationships	82.9 ± 6.0 [16]	82.7 ± 5.8 [7]	83.1 ± 6.1 [9]	Mean D = 0.43 (−0.39, 1.25)	0.304
Environmental	78.4 ± 5.9 [16]	78.2 ± 5.6 [7]	78.5 ± 6.1 [9]	Mean D = 0.35 (−0.45, 1.16)	0.391
Physical	74.7 ± 6.1 [16]	74.6 ± 6.3 [7]	74.7 ± 5.9 [9]	Mean D = 0.12 (−0.71, 0.95)	0.780
Psychological	82.1 ± 5.6 [16]	81.9 ± 5.7 [7]	82.2 ± 5.5 [9]	Mean D = 0.22 (−0.54, 0.98)	0.565

Data are number (%), mean ± SD, or median (95% confidence interval). Numbers in square brackets indicate patients with missing data

*PACU* post-anesthesia care unit

^a^Calculated as emergence delirium vs. or minus no emergence delirium

^b^Assessed with the Telephone Interview for Cognitive Status-modified; score ranges from 0 to 50, with higher score indicating better function

^c^Assessed with the World Health Organization Quality of Life-brief version; scores range from 0 to 100 in each domain, with higher score indicating better quality of life

### Association between emergence delirium and postoperative delirium

After correction for confounding factors, emergence delirium was independently associated with an increased risk of POD (OR 1.717, 95% CI 1.078–2.735, *P* = 0.007) (Table 3 and Table 4).

**Table 3:** Univariate analysis (Logistic regression model)

	Postoperative delirium		Emergence delirium	
	Odds ratio (95% CI)	*P* value	Odds ratio (95% CI)	*P* value
Age (years)	1.071 (1.032–1.110)	<0.001	1.077 (1.049–1.105)	< 0.001
Male sex	0.903 (0.597–1.365)	0.627	0.807 (0.614–1.060)	0.124
Body mass index (kg/m^2^)	0.944 (0.889–1.002)	0.058	0.986 (0.948–1.025)	0.473
Education (year)	0.984 (0.942–1.028)	0.474	0.945 (0.917–0.973)	< 0.001
Preoperative comorbidity				
Stroke	2.514 (1.273–4.964)	0.008	0.743 (0.406–1.360)	0.336
Hypertension	0.841 (0.559–1.265)	0.406	0.946 (0.723–1.237)	0.683
Coronary heart disease	1.030 (0.576–1.844)	0.919	1.468 (1.008–2.139)	0.046
Arrhythmia	1.729 (0.846–3.536)	0.133	1.254 (0.728–2.160)	0.415
Pulmonary disease	2.043 (1.073–3.892)	0.030	1.456 (0.880–2.411)	0.144
Diabetes	1.264 (0.799–1.999)	0.317	1.076 (0.787–1.472)	0.646
Abnormal liver function	2.703 (1.325–5.515)	0.006	1.256 (0.684–2.306)	0.462
Malignant tumor	1.742 (1.000–3.033)	0.050	1.005 (0.660–1.530)	0.983
Charlson Comorbidity Index (point)	1.104 (1.024–1.190)	0.010	1.016 (0.961–1.073)	0.580
ASA classification III (vs. II)	2.065 (1.363–3.128)	0.001	1.434 (1.065–1.930)	0.018
Chronic smoking	1.164 (0.733–1.848)	0.520	0.739 (0.536–1.018)	0.064
Alcoholism	0.815 (0.450–1.475)	0.499	0.737 (0.504–1.080)	0.117
History of surgery	1.439 (0.947–2.186)	0.088	1.382 (1.054–1.813)	0.019
Preoperative lab tests				
Hematocrit (%)	0.932 (0.898–0.967)	< 0.001	0.951 (0.927–0.975)	<0.001
Albumin (g/L)	0.957 (0.919–0.996)	0.030	0.956 (0.929–0.984)	0.002
Creatinine (μmol/L)	1.005 (0.996–1.014)	0.288	1.003 (0.997–1.009)	0.354
Na^+^<135.0 or > 145.0 mmol/L	0.686 (0.207–2.273)	0.537	0.916 (0.460–1.823)	0.802
K^+^ < 3.5 or > 5.5 mmol/L	0.887 (0.469–1.675)	0.711	1.210 (0.814–1.797)	0.346
Preoperative MMSE (score)	0.858 (0.792-0.930)	< 0.001	0.791 (0.743-0.841)	<0.001
Duration of anesthesia (min)	1.004 (1.002–1.006)	0.001	1.002 (1.000–1.003)	0.075
General anesthesia (vs. combined)	1.519 (1.051–2.197)	0.026	0.939 (0.736–1.200)	0.616
Intraoperative medication				
Use of nitrous oxide	1.054 (0.693–1.602)	0.807	1.150 (0.873–1.516)	0.319
Use of sevoflurane	1.071 (0.693–1.656)	0.757	1.206 (0.902–1.607)	0.201
Use of dexmedetomidine	0.645 (0.423–0.981)	0.040	1.092 (0.835–1.429)	0.520
Use of midazolam	1.325 (0.823–2.133)	0.246	0.865 (0.618–1.210)	0.396
Use of etomidate	1.435 (0.851–2.421)	0.175	1.456 (1.050–2.020)	0.024
Propofol (mg)	1.001 (1.000–1.001)	0.039	1.000 (1.000–1.001)	0.048
Remifentanil (μg)	1.000 (1.000–1.000)	0.578	1.000 (1.000–1.000)	0.819
Sufentanil (μg)	1.007 (1.002–1.012)	0.012	1.002 (0.998–1.006)	0.391
Sufentanil equivalent (μg)	1.001 (0.998–1.004)	0.488	1.000 (0.998–1.002)	0.853
Rocuronium (mg)	1.011 (1.000–1.023)	0.052	1.000 (0.993–1.007)	0.914
NSAIDs	1.186 (0.702–2.006)	0.524	1.091 (0.781–1.523)	0.609
Total fluid infusion (ml)	1.000 (1.000–1.000)	0.002	1.000 (1.000–1.000)	<0.001
Allogeneic blood transfusion	1.613 (0.856–3.038)	0.139	2.179 (1.369–3.469)	0.001
Estimated blood loss (ml)	1.000 (1.000–1.001)	0.111	1.001 (1.000–1.001)	0.002
Lowest intraoperative Hb < 10 g/dL	2.446 (1.366–4.379)	0.003	2.454 (1.529–3.939)	<0.001
Duration of surgery (min)	1.003 (1.001–1.006)	0.010	1.002 (1.000–1.004)	0.078
Thoraco-/laparoscopic surgery	0.790 (0.524–1.192)	0.262	1.593 (1.213–2.093)	0.001
Location of surgery				
Intra-thoracic	1.278 (0.678–2.407)	0.448	1.350 (0.952–1.915)	0.092
Intra-abdominal	1.129 (0.654–1.947)	0.663	1.643 (1.081–2.497)	0.020
Spinal/extremities/others	Ref		Ref	
Emergence agitation	1.478 (1.185-1.843)	0.001	5.515 (4.515–6.737)	< 0.001
Temperature at PACU admission	0.930 (0.585–1.479)	0.760	0.437 (0.316–0.604)	< 0.001
Temperature < 36 °C	0.990 (0.640–1.529)	0.963	2.429 (1.828–3.228)	< 0.001
First NRS pain score in PACU	0.911 (0.802–1.036)	0.157	1.129 (1.041–1.224)	0.003
Average NRS pain score in PCAU	0.992 (0.866–1.137)	0.912	1.255 (1.146–1.375)	<0.001
Use of patient-controlled analgesia	3.775 (1.777–8.021)	0.001	1.286 (0.780–2.118)	0.324
Supplemental NSAIDs within 5 days	1.264 (0.781–2.046)	0.340	–	–
Supplemental opioids within 5 days	1.692 (0.800–3.580)	0.169	–	–
Emergence delirium	2.275 (1.506–3.436)	< 0.001	–	–
First NRS pain score in wards	1.412 (1.242–1.606)	< 0.001	–	–
Average NRS pain score in wards	1.534 (1.327–1.773)	< 0.001	–	–
Postoperative complications in 5 days	4.415 (2.411–7.124)	< 0.001	–	**–**

*ASA* American Society of Anesthesiologists, *PACU* post-anesthesia care unit, *NRS* numeric rating scale, *NSAIDs* non-steroid anti-inflammatory drugs

**Table 4:** Predictors of postoperative delirium (Logistic regression model)

Factors	Univariable analyses		Multivariable analysis^a^	
	Odds ratio (95% CI)	*P* value	Odds ratio (95% CI)	*P* value
Emergence delirium	2.275 (1.506–3.436)	< 0.001	1.717 (1.078–2.735)	0.023
Age (year)	1.071 (1.032–1.110)	< 0.001	1.048 (1.004–1.093)	0.031
Body mass index (kg/m^2^)	0.944 (0.889–1.002)	0.058	0.965 (0.904–1.031)	0.293
Charlson Comorbidity Index (unit)	1.104 (1.024-–1.190)	0.010	1.058 (0.968–1.155)	0.215
History of surgery	1.439 (0.947–2.186)	0.088	1.268 (0.784–2.049)	0.334
Preoperative hematocrit (%)	0.932 (0.898–0.967)	< 0.001	0.951 (0.906–0.998)	0.042
Preoperative albumin (g/L)	0.957 (0.919–0.996)	0.030	1.037 (0.981–1.096)	0.196
Preoperative MMSE (score)	0.858 (0.792–0.930)	< 0.001	0.885 (0.809–0.969)	0.008
General anesthesia	1.519 (1.051–2.197)	0.026	1.147 (0.713–1.845)	0.571
Use of dexmedetomidine	0.645 (0.423–0.981)	0.040	0.658 (0.413–1.047)	0.077
Use of midazolam	1.325 (0.823–2.133)	0.246	1.277 (0.751–2.171)	0.367
Use of etomidate	1.435 (0.851–2.421)	0.175	1.524 (0.849–2.739)	0.158
Allogeneic blood transfusion	1.613 (0.856–3.038)	0.139	1.038 (0.495–2.176)	0.922
Duration of surgery (min)	1.003 (1.00–1.006)	0.010	1.002 (0.998–1.005)	0.329
Supplemental opioids within 5 days	1.692 (0.800–3.580)	0.169	1.403 (0.774–2.542)	0.264
Use of patient-controlled analgesia	3.775 (1.777–8.021)	0.001	2.984 (1.035–8.608)	0.043
Average NRS pain score in wards	1.534 (1.327–1.773)	< 0.001	1.579 (1.347–1.852)	<0.001
Postoperative complications in 5 days	4.415 (2.411–7.124)	< 0.001	3.422 (1.834–6.384)	<0.001

*MMSE* mini-mental status examination, *NRS* numeric rating scales

^a^Variables with *P* < 0.20 in univariable analyses and those that were considered clinically important were included in the multivariable Logistic regression model (Enter). Preoperative comorbidities including stroke, arrhythmia, pulmonary disease, abnormal liver function, malignant tumor, and ASA classification (III vs. II) were excluded due to correlation with Charlson Comorbidity Index. Duration of anesthesia, propofol (mg), sufentanil (μg), rocuronium (mg), and total fluid infusion (ml) were excluded due to correlation with duration of surgery. Emergence agitation was excluded due to correlation with emergence delirium. Estimated blood loss (ml) and the lowest intraoperative Hb < 10 g/dL were excluded due to correlation with allogeneic blood transfusion. First NRS pain score in wards was excluded due to correlation with average NRS pain score in the wards. Hosmer–Lemeshow test of the multivariable model: χ^2^ = 7.116, df = 8, *P* = 0.524

### Risk factors of emergence delirium

The results showed that advanced age (OR 1.042, 95% CI 1.003–1.083, *P* = 0.037), occurrence of emergence agitation (OR 6.007, 95% CI 4.752–7.595, *P* < 0.001), hypothermia at PACU admission (OR 2.672, 95% CI 1.790–3.988, *P* < 0.001), and higher average NRS pain score in PACU (OR 1.309, 95% CI 1.150–1.489, *P* < 0.001) were associated with a higher risk; whereas male sex (OR 0.530, 95% CI 0.332–0.846, *P* = 0.008), higher preoperative albumin level (OR 0.953, 95% CI 0.912–0.997, *P* = 0.036) and higher preoperative MMSE score (OR 0.799, 95% CI 0.727–0.878, *P* < 0.001) were associated with a lower risk of emergence delirium (Tables 3 and 5).

**Table 5:** Predictors of emergence delirium (Logistic regression model)

Factors	Univariate analysis		Multivariate analysis^a^	
	Odds ratio (95% CI)	*P* value	Odds ratio (95% CI)	*P* value
Age (year)	1.077 (1.049–1.105)	< 0.001	1.042 (1.003–1.083)	0.037
Male sex	0.807 (0.614–1.060)	0.124	0.530 (0.332–0.846)	0.008
Education (year)	0.945 (0.917–0.973)	< 0.001	0.996 (0.952–1.043)	0.872
ASA classification III	1.434 (1.065–1.930)	0.018	1.291 (0.830–2.006)	0.257
Chronic smoking	0.739 (0.536–1.018)	0.064	0.651 (0.373–1.137)	0.131
Alcoholism	0.737 (0.504–1.080)	0.117	0.929 (0.485–1.780)	0.824
History of surgery	1.382 (1.054–1.813)	0.019	1.054 (0.712–1.559)	0.792
Preoperative hematocrit (%)	0.951 (0.927–0.975)	< 0.001	0.993 (0.953–1.036)	0.761
Preoperative albumin (g/L)	0.956 (0.929–0.984)	0.002	0.953 (0.912–0.997)	0.036
Preoperative MMSE (score)	0.791 (0.743–0.841)	< 0.001	0.799 (0.727–0.878)	< 0.001
Use of sevoflurane	1.206 (0.902–1.607)	0.201	1.009 (0.676–1.508)	0.963
Use of etomidate	1.456 (1.050–2.020)	0.024	1.263 (0.803–1.987)	0.313
Allogeneic blood transfusion	2.179 (1.369–3.469)	0.001	1.696 (0.867–3.318)	0.123
Duration of surgery (min)	1.002 (1.000–1.004)	0.078	1.000 (0.997–1.003)	0.949
Emergence agitation	5.515 (4.515–6.737)	< 0.001	6.007 (4.752–7.595)	< 0.001
Thoraco-/laparoscopic surgery	1.593 (1.213–2.093)	0.001	0.807 (0.543–1.199)	0.288
Hypothermia at PACU admission^b^	2.429 (1.828–3.228)	< 0.001	2.672 (1.790–3.988)	< 0.001
Average NRS pain score in PACU	1.255 (1.146–1.375)	< 0.001	1.309 (1.150–1.489)	< 0.001
Use of patient-controlled analgesia	1.286 (0.780–2.118)	0.324	0.946 (0.455–1.969)	0.882

*MMSE* mini-mental status examination, *PACU* post-anesthesia care unit

^a^Variables with *P* < 0.20 in univariable analyses and those that were considered clinically important were included in the multivariable Logistic regression model (Enter). Preoperative comorbidities including coronary heart disease and pulmonary disease were excluded due to correlation with ASA classification (III vs. II). Duration of anesthesia, propofol (mg) and total fluid infusion (ml) were excluded due to correlation with duration of surgery. Estimated blood loss (ml) and lowest intraoperative Hb < 10 g/dL were excluded due to correlation with allogeneic blood transfusion. Temperature at PACU admission was excluded due to correlation with temperature at PACU admission < 36 °C. First NRS pain score in PACU was excluded due to correlation with average NRS pain score in PACU. Hosmer–Lemeshow test of the multivariable model: χ2 = 17.990, df = 8, P = 0.021

^b^Tympanic temperature < 36 °C at PACU admission

## Discussion

Our observational study of elderly patients showed that, after general anesthesia and major noncardiac surgery, 37.0% developed emergence delirium during PACU stay and 11.4% developed POD. The occurrence of emergence delirium is independently associated with an increased risk of the POD development. Patients with emergence delirium stayed longer in the PACU and hospital after surgery, and developed more non-delirium complications within 30 days.

In the present study, the prevalence of emergence delirium was higher than those reported previously [4–13]. The reasons for this discrepancy may include the followings. Firstly, patients included in the present study were at higher risk. For example, most previous studies included all the types of surgery with any age patients [4–13], whereas our patients were older and underwent longer surgeries. All of which might have increased emergence delirium [4, 5, 9, 12, 13]. Secondly, the delirium assessment methods and timepoints varied in available studies. In fact, some of the previous studies assessed agitation rather than delirium. For example, emergence delirium was defined when the Riker sedation–agitation scale was ≥ 5 or the RASS was ≥+1 or higher [4, 5, 7, 9, 10, 13]. As shown by our results and others, the majority of emergence delirium has a hypoactive phenotype [6]. Thus, those studies might have missed hypoactive delirium and underestimated the incidence. To be noted, emergence delirium often occurs early after anesthesia. When it was assessed at PACU admission in the elderly, the reported prevalence (31–45%) was similar as ours [6, 14, 15]. The incidence of POD in our results was well within the range as expected [16–18].

The impact of emergence delirium on the occurrence of POD has not been fully investigated. In an observational study, Xara et al. [5] reported that POD was more common in patients with emergence delirium (defined as a RASS score ≥+1) or hypoactive emergence (defined as a RASS score ≤ −2). In a recent retrospective case-controlled study, Fields et al. [22] reported that the incidence of POD was significantly higher in patients with emergence agitation (defined as a RASS score ≥+3 or required haloperidol). In two small sample size studies of elderly patients, emergence delirium in the PACU was also found to be associated with POD [14, 15]. In the present study of sufficient sample size, we assessed both emergence delirium and POD according to the consensus published recently [3]. Our results showed that, after adjustment with confounding factors, the occurrence of emergence delirium was independently associated with an increased risk of POD in the elderly after general anesthesia and major surgery. Furthermore, we found that patients with emergence delirium stayed longer in the PACU and hospital and developed more non-delirium complications within 30 days; which were in line with previous studies [5, 8–10, 22]. Therefore, delirium monitoring should be performed PACU patients.

The potential mechanisms underlying the association between emergence delirium and POD are not clear. According to previous studies, most of the risk factors of emergence delirium are similar with those of POD. For example, among the identified predictors of emergence delirium, patient-related factors include old age, pre-existing diseases, substance misuse, cognitive impairment, and psychiatric problems; treatment-related factors include premedication with benzodiazepine, etomidate for induction, EEG burst suppression during anesthesia, major and long-duration surgery, large blood loss, and high pain score after surgery [4–13]. In the present study, old age, low albumin level, poor cognitive function, and high pain score were identified as risk factors of emergence delirium; which were in accord with previous studies [4–13]. And all these factors have been reported to be associated increased odds of POD [16–18]. The existence of common risk factors indicates that emergence delirium may share some similar mechanisms with POD but warrants further study. It is noteworthy that emergence agitation was identified as a risk factor of emergence delirium. As a matter of fact, the two terms have been used interchangeably in previous studies, and the same assessment tool (e.g., RASS or Riker Sedation-Agitation Scale) have been used for both conditions [4, 5, 9, 10, 13, 34]. We also found that hypothermia at the PACU admission was associated with emergence delirium development. A similar result was reported by others for emergence delirium but not POD [5]. The above results suggest that maintaining normothermia during anesthesia, preventing agitation during emergence and providing optimal analgesia may reduce emergence delirium and subsequent postoperative delirium but requires further demonstration. Contrary to some of the previous results [34], we found that male patients developed less emergence delirium; more studies are needed.

The strengths of the present study include a relatively large sample size and strictly assessed delirium. There are some limitations. Firstly, as an observational study, we cannot establish a causal relationship between emergence delirium and POD. The clinical impact of emergence delirium needs to be studied further. Secondly, as a single center study, the generalizability of our results may be limited. Thirdly, whether emergence delirium is associated with worse long-term outcomes remains unclear and requires further study.

## Conclusions

Our results showed that, in elderly patients admitted to the PACU after general anesthesia and major surgery, emergence delirium is common and is independently associated with an increased risk of postoperative delirium. Patients with emergence delirium have worse perioperative outcomes. Whether measures effective in preventing emergence delirium can reduce postoperative delirium and improve perioperative outcome need to be studied further.

## Electronic supplementary material

Below is the link to the electronic supplementary material.

Supplementary material 1 (DOCX 31 kb)
