# Supplementary material for: Emergence delirium is associated with increased postoperative delirium in elderly: a prospective observational study
Source: J Anesth. 2020 Jun 7;34(5):675–87. doi: 10.1007/s00540-020-02805-8 (PMC7511467; doi:10.1007/s00540-020-02805-8)
Supplement: Supplementary file 1 — Supplementary material 1 (DOCX 31 kb) [file 540_2020_2805_MOESM1_ESM.docx]

Supplemental Table 1. Individual non-delirium complications within 5 days after surgery

|  | Total (n=915) | Emergence delirium (n=339) | No emergence delirium (n=576) | P value |
| --- | --- | --- | --- | --- |
| Stroke ^a^ | 6 (0.7%) | 3 (0.9%) | 3 (0.5%) | 0.510 |
| Acute coronary syndrome ^b^ | 20 (2.2%) | 11 (3.2%) | 9 (1.6%) | 0.093 |
| Arrhythmia ^c^ | 23 (2.5%) | 12 (3.5%) | 11 (1.9%) | 0.128 |
| Circulatory insufficiency ^d^ | 18 (2.0%) | 7 (2.1%) | 11 (1.9%) | 0.870 |
| Respiratory failure ^e^ | 15 (1.6%) | 6 (1.8%) | 9 (1.6%) | 0.811 |
| Renal failure ^f^ | 2 (0.2%) | 1 (0.3%) | 1 (0.2%) | 0.704 |
| Acute kidney injury ^g^ | 23 (2.5%) | 7 (2.1%) | 16 (2.8%) | 0.506 |
| Surgical bleeding ^h^ | 3 (0.3%) | 0 (0.0%) | 3 (0.5%) | 0.183 |
| Anastomotic leakage | 10 (1.1%) | 7 (2.1%) | 3 (0.5%) | **0.030** |
| Gastrointestinal bleeding ^i^ | 6 (0.7%) | 3 (0.9%) | 3 (0.5%) | 0.510 |
| Severe sepsis ^j^ | 9 (1.0%) | 5 (1.5%) | 4 (0.7%) | 0.248 |
| Surgical-site infection ^k^ | 12 (1.3%) | 6 (1.8%) | 6 (1.0%) | 0.350 |
| Death | 2 (0.2%) | 1 (0.3%) | 1 (0.2%) | 0.704 |

Data are number (%).

^a^ Persisted new focal neurologic deficit and confirmed by neurologic imaging.

^b^ Included acute myocardial infarction and unstable angina, which were confirmed by clinical symptoms, electrocardiographic changes and serum cardiac troponin I concentration. Diagnoses were confirmed by cardiologists.

^c^ Includes new onset atrial fibrillation, II/III degree atrioventricular block, premature ventricular contractions (bigeminy/trigeminy) and ventricular tachycardia, which required medical or interventional therapy.

^d^ Requirement of inotropics or vasopressors for ≥24 hours.

^e^ Arterial blood partial pressure of oxygen (PaO2) <60 mmHg on room air, a ratio of PaO2 to inspired oxygen fraction <300 or pulse oxygen saturation <90%, and requirement of oxygen therapy or mechanical ventilation.

^f^ Requirement of renal replacement therapy.

^g^ Diagnosed according to the KDIGO criteria [31].

^h^ Requirement of secondary surgical hemostasis.

^i^ Positive occult blood test results in gastric contents or stool with decreased hemoglobin, and required blood transfusion.

^j^ Two or more signs of systematic inflammatory response syndrome caused by infection, with at least one new organ/system dysfunction or required vasopressors to maintain blood pressure.

^k^ Required further debridement or drainage.

Supplemental Table 2. Individual non-delirium complications within 30 days after surgery

|  | Total (n=915) | Emergence delirium (n=339) | No emergence delirium (n=576) | P value |
| --- | --- | --- | --- | --- |
| Stroke ^a^ | 6 (0.7%) | 3 (0.9%) | 3 (0.5%) | 0.510 |
| Acute coronary syndrome ^b^ | 20 (2.2%) | 11 (3.2%) | 9 (1.6%) | 0.093 |
| Arrhythmia ^c^ | 25 (2.7%) | 13 (3.8%) | 12 (2.1%) | 0.117 |
| Circulatory insufficiency ^d^ | 20 (2.2%) | 9 (2.7%) | 11 (1.9%) | 0.457 |
| Respiratory failure ^e^ | 16 (1.7%) | 6 (1.8%) | 10 (1.7%) | 0.970 |
| Renal failure ^f^ | 2 (0.2%) | 1 (0.3%) | 1 (0.2%) | 0.704 |
| Acute kidney injury ^g^ | 26 (2.8%) | 9 (2.7%) | 17 (3.0%) | 0.794 |
| Surgical bleeding ^h^ | 3 (0.3%) | 0 (0.0%) | 3 (0.5%) | 0.183 |
| Anastomotic leakage | 11 (1.2%) | 7 (2.1%) | 4 (0.7%) | 0.066 |
| Gastrointestinal bleeding ^i^ | 7 (0.8%) | 4 (1.2%) | 3 (0.5%) | 0.296 |
| Delayed oral intake ^j^ | 18 (1.9%) | 12 (3.3%) | 6 (1.0%) | **0.009** |
| Severe sepsis ^k^ | 9 (1.0%) | 5 (1.5%) | 4 (0.7%) | 0.248 |
| Surgical-site infection ^l^ | 16 (1.7%) | 8 (2.4%) | 8 (1.4%) | 0.279 |
| Death | 11 (1.2%) | 6 (1.8%) | 5 (0.9%) | 0.227 |

Data are number (%).

^a^ Persisted new focal neurologic deficit and confirmed by neurologic imaging.

^b^ Included acute myocardial infarction and unstable angina, which were confirmed by clinical symptoms, electrocardiographic changes and serum cardiac troponin I concentration. Diagnoses were confirmed by cardiologists.

^c^ Includes new onset atrial fibrillation, II/III degree atrioventricular block, premature ventricular contractions (bigeminy/trigeminy) and ventricular tachycardia, which required medical or interventional therapy.

^c^ Requirement of inotropics or vasopressors for ≥24 hours.

^e^ Arterial blood partial pressure of oxygen (PaO2) <60 mmHg on room air, a ratio of PaO2 to inspired oxygen fraction <300 or pulse oxygen saturation <90%, and requirement of oxygen therapy or mechanical ventilation.

^f^ Requirement of renal replacement therapy.

^g^ Diagnosed according to the KDIGO criteria [31].

^h^ Requirement of secondary surgical hemostasis.

^i^ Positive occult blood test results in gastric contents or stool with decreased hemoglobin, and required blood transfusion.

^j^ Requirement of intravenous nutrition for >1 week due to enteroparalysis, ileus, or other complications.

^k^ Two or more signs of systematic inflammatory response syndrome caused by infection, with at least one new organ/system dysfunction or required vasopressors to maintain blood pressure.

^l^ Required further debridement or drainage.
